# Game‐Based Learning: A Pilot Educational Strategy to Foster the Learning of Endodontic Terminology

**DOI:** 10.1111/iej.70001

**Published:** 2025-07-18

**Authors:** Isabel Fernandez‐Garcia, Paula Riaza, Ana Arias

**Affiliations:** ^1^ Department of Conservative and Prosthetic Dentistry, School of Dentistry Complutense University Madrid Spain

**Keywords:** educational gaming, endodontic games, game‐based learning, gamingdental education

## Abstract

**Aim:**

Exploring the effectiveness of a game‐based pilot educational strategy for learning endodontic terminology and assessing satisfaction among postgraduate students.

**Methodology:**

The study was conducted on the first‐ and second‐year students of the postgraduate program in Endodontology (*n* = 12). A game‐based activity, modelled on a television quiz show, was designed to facilitate the learning of endodontic terminology. Students were randomly allocated into four teams based on their course year. Each team answered 26 questions corresponding to definitions of endodontic terms, with correct answers beginning with or containing consecutive letters of the alphabet. Teams within the same cohort competed, and the team with the most correct answers was declared the winner. Students completed three tests at different time points (2 weeks, 1 month and 1 year) after the game‐based educational session. A trained rater blindly scored the tests. Additionally, participants completed an anonymous satisfaction web‐based survey. The survey included multiple‐choice questions to be answered with a 5‐point Likert scale to address satisfaction with the content and instructional methods and four questions assessing overall satisfaction on a scale from 0 to 10, along with open‐ended questions for additional feedback. Scores of students and numerical responses in the satisfaction survey were compared between first‐ and second‐year students with the Mann–Whitney *U* test. Scores obtained 2 weeks and 1 month/1 year after the instructional activity were compared with the Wilcoxon paired test. Responses to the multiple‐choice questions, based on a 5‐point Likert scale, regarding the impact of content and instructional methods were compared between first‐ and second‐year students with the ordinal chi‐squared test.

**Results:**

Students demonstrated high test scores and reported high satisfaction with the game‐based learning strategy. No statistically significant differences in test scores or satisfaction levels were observed between cohorts. Additionally, test scores remained consistent regardless of the time elapsed between the activity and testing (*p* > 0.05).

**Conclusions:**

The high scores and satisfaction reported by both cohorts suggest that game‐based educational strategies are an innovative practical approach that may serve as an additional method to memorise endodontic terminology.

## 1 Introduction

Lectures and master classes remain among the most widely used educational methods due to their efficiency in delivering large volumes of information to a broad audience. This approach can reduce cognitive load by organising content in a structured and systematic manner (Charlton 2006; Dietrich and Evans 2022; Kay et al. 2019). However, learners often struggle to maintain active attention during the lecture (Davis 2009), particularly Generation Z students (the majority of learners at dental schools), who prefer personalised and innovative learning methods over passive instruction (Ismail and Mohammad 2017), as well as technology‐enhanced and interactive learning environments (Eckleberry‐Hunt et al. 2018; Shorey et al. 2021) At the same time, while digital fluency may facilitate learning, excessive use of mobile devices has also been associated with reduced attention span and academic performance (Siyami et al. 2023).

Research suggests that active student engagement enhances understanding and can positively impact knowledge acquisition (Zheng and Ferreira 2021). Active learning is defined as any instructional method that requires students to meaningfully engage with activities while reflecting on their actions (Bonwell and Eison 1991). This is particularly effective in small‐group settings, like postgraduate programmes with a limited number of students, where it promotes active student participation and fosters a collaborative partnership between professors/tutors and students in the educational process (Plasschaert et al. 2007).

In parallel, key principles of andragogy suggest that adult learners benefit most from instructional approaches that are self‐directed, experience‐based, goal‐oriented and intrinsically motivating. Within this framework, gamification has emerged as a suitable instructional method that supports autonomy, motivation and meaningful learning (Bigdeli et al. 2023; Plass et al. 2015). Gamification is defined as the use of design elements characteristic of games in non‐game contexts (Deterding et al. 2011). Unlike games, gamification is driven by a more serious objective. It involves the introduction of game‐like aspects to non‐game tasks and contexts. Game‐based learning and serious games, on the other hand, refer to the application of full‐featured games (Krath et al. 2021). The use of different game‐based strategies adapted for educational activities has been implemented in dental education, positively impacting learning outcomes (Felszeghy et al. 2019; Nguyen et al. 2023; Sharmin and Chow 2023). In particular, game‐based activities improved motivation (Griffith et al. 1983; Perez et al. 2023; Sharmin and Chow 2023), a fundamental precondition for effective learning as supported by constructivist learning theories (Krath et al. 2021; Rohman and Fauziati 2022). At the same time, several studies have reported that gamification is as efficient as other methods in achieving educational goals, allowing for the review and integration of knowledge through fun and engaging hands‐on activities (Cooperride and Whitney 2000; Castro et al. 2019). Additionally, game‐based strategies foster interactive learning environments that engage learners effectively while enhancing their knowledge, motivation and satisfaction (Cooperride and Whitney 2000; Ferreri and O'Connor 2013). Students prefer collaborative learning (Godwin‐Jones 2014), and the open design of gaming strategies may accommodate this preference. All these benefits are particularly relevant in postgraduate education, where students are typically adults and digital native learners who may benefit from more autonomous and interactive approaches (Liu et al. 2020), although it must be acknowledged that students integrate knowledge in different ways and not all students may benefit the same from active learning strategies (Cassidy 2010).

At the same time, learning endodontic terminology can be a challenging task for students. While terminology acquisition is considered the lowest level of Bloom's taxonomy, it serves as the foundation for the development of higher‐order cognitive processes (Ormell 1974; Adams 2015). In postgraduate education, mastery of domain‐specific vocabulary is essential for effective clinical communication and advance learning; however, traditional memorization techniques using strategies like recitation can lead to boredom or anxiety and are associated with reduced long‐term retention (Blondé et al. 2022; Urrizola et al. 2023). In contrast, game‐based strategies for vocabulary learning have been shown to reduce anxiety while increasing student motivation and interest (Griffith et al. 1983; Wei et al. 2018; Zhonggen 2018; Zeybek and Saygı 2024).

Therefore, the aim of this pilot study was to assess the effectiveness of a game‐based educational strategy for learning endodontic terminology and evaluate the satisfaction among postgraduate students.

## 2 Materials and Methods

Approval for the study (CE_20230413‐03_SAL) was sought from the Institutional Review Board (IRB) of Complutense University, and exemption was granted to allow the study to be carried out on first‐ and second‐year students on the postgraduate programme in Endodontology.

### 2.1 Participants

The programme accepts six students per year. The 12 postgraduate students were invited to participate in the study and signed an informed consent. The six students in each academic year accepted to participate and were randomly separated into two groups of three students each. That makes a total of four groups with three students each (two with first year and two with second‐year students).

### 2.2 Learning Strategy

A game‐based activity, modelled on a television quiz show, was designed to help students learn endodontic terminology. The strategy was inspired by a television (TV) game show format known by different names across various European countries: *Alphabetical* in the United Kingdom, *Pasapalabra* in Spain or *Passaparola* in Italy. The original version, *The Alphabet Game*, was a comedy panel game show that aired on BBC One from 5 August 1996 to 27 March 1997. It was later revived in the United Kingdom as *Alphabetical* (2016–2017), whereas the Spanish adaptation, *Pasapalabra*, has remained popular and continues to air to this day (https://en.wikipedia.org/wiki/The_Alphabet_Game). The present study replicated the final round of the TV game show, known as *El Rosco* in the Spanish version.

Contestants compete by answering separate sets of 26 questions, each corresponding to a different letter of the alphabet from A to Z. The answer to each question begins or contains the given letter. Only one contestant plays at a time, using time from their individual clock, while the opponent's clock remains paused. Both begin at the letter A and proceed sequentially through the alphabet. If a contestant passes or gives an incorrect answer, control shifts to the opponent, who resumes play from the letter following the one that they last missed or passed. A contestant may only revisit a previously passed question after completing the full sequence of letters and returning to the original prompt. Play continues until a contestant either answers all 26 questions or exhausts their allotted time. If one contestant runs out of time, the other continues alone. The first contestant to correctly answer all 26 questions wins a progressive jackpot. If neither contestant wins the jackpot, the contestant with the most correct answers wins while the jackpot carries over to the next episode. In the Spanish version, they can go so long without a winner that the rollover jackpot sometimes reaches seven figures.

Based on this structure of this game, two separate groups from the same academic cohort competed by answering distinct sets of 26 questions. Each set included one endodontic term corresponding to a different letter of the alphabet, from A to Z, presented in sequential order. The target term for each question either began with or contained the designated letter.

An instructor (IF) prepared the 104 questions by consulting endodontic terminology in the Tenth Edition of the American Association of Endodontists (AAE) (n.d.) Glossary of Endodontic Terms, updated in 2020 (AAE, *Glossary of Endodontic Terms*) and translating to Spanish. If no suitable term was available for a particular letter, the last name of relevant authors in the field of endodontology was included in the set of questions. For each letter of the alphabet, four terms and definitions were selected, classified by level of difficulty and randomly assigned to build four different question sets (two easier and two complex). Four set of questions (one for each team), from A to Z, were thus prepared. As examples the easier set contained definitions like ‘A tear‐drop shape that may be formed in the apical foramen during preparation of a curved canal when a file extends through the foramen and transports the outer wall’ and the more complex others like ‘The condition when a portion of the pulp has become infected and necrosed and the remaining pulp is inflamed’. The easier sets were used with first‐year students, whereas the complex sets were assigned to second‐year students.

### 2.3 Intervention

Students were not informed of the activity beforehand to ensure that no prior preparation or study could influence the outcome. To maintain the element of surprise and avoid drawing attention to the activity, a separate set of lectures was scheduled for the session. Upon arrival in the classroom, students were invited to participate and were presented with an informed consent form outlining the nature of the activity and the potential use of anonymized aggregated data for publication. The rules of the game were then explained, and the activity was carried out after students had voluntarily agreed to participate and demonstrated understanding of how the game worked.

Students in the same cohort were then randomly allocated to different groups. The two teams composed of first‐year students played first. Each team was given a maximum of 2 min to answer the set of questions. A coin toss determined which team would start.

**FIGURE 1:**
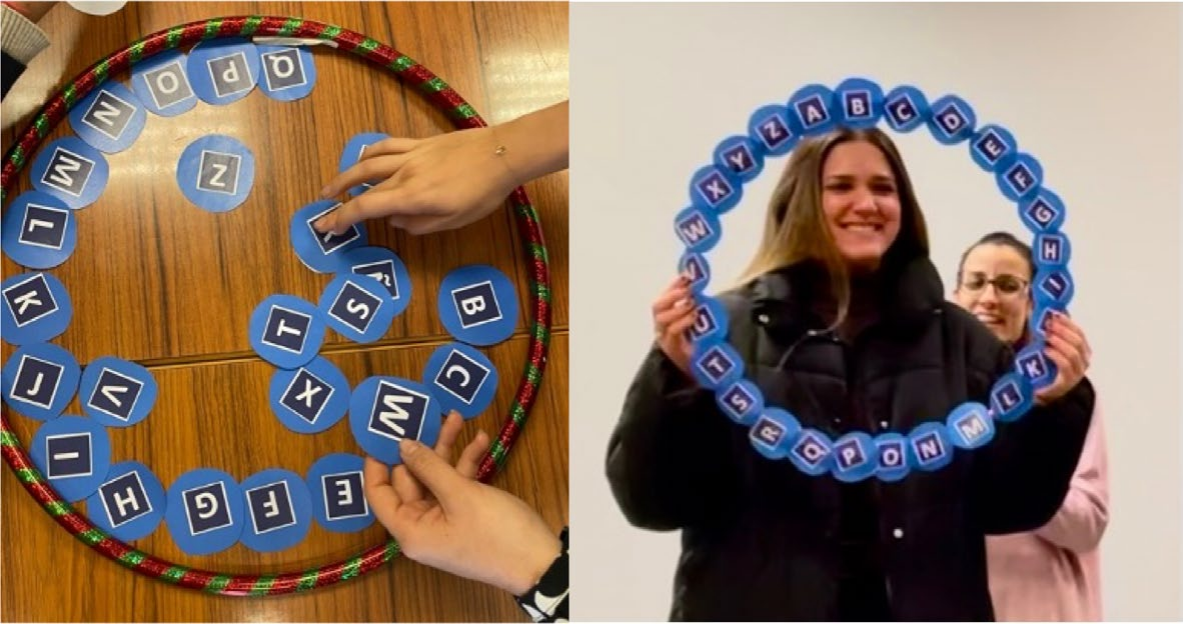
Image of the construction of the hula‐hoop with the letters of the alphabet and the use in the educational session.

**FIGURE 2:**
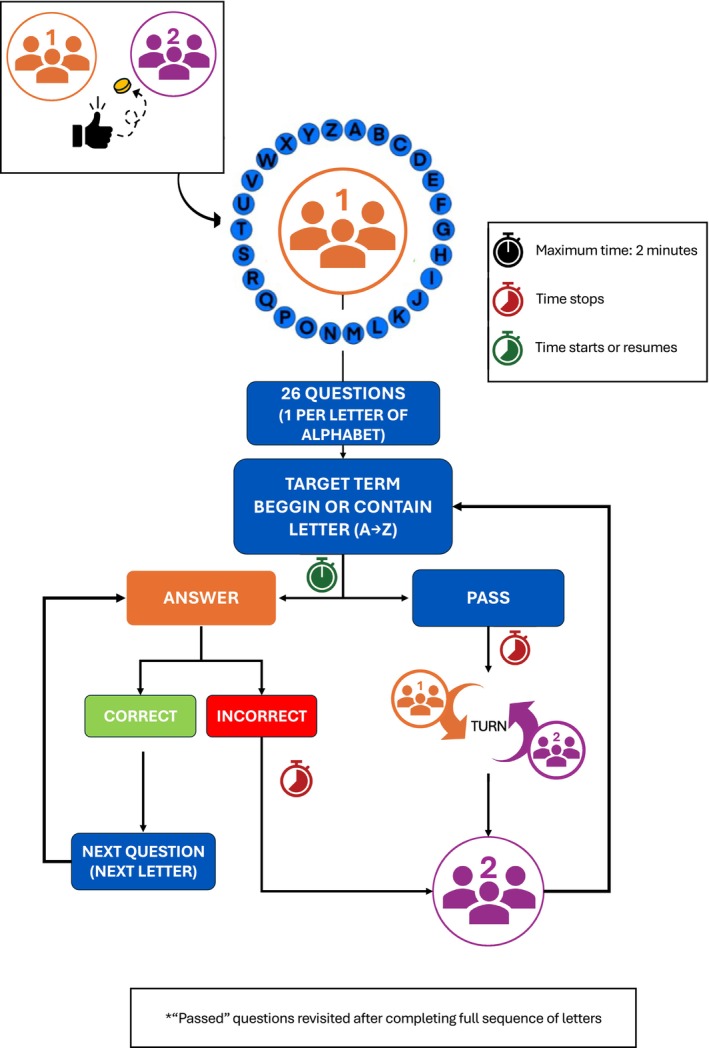
Schematic representation of the game‐based educational activity.

Teams alternated turns, with one contestant representing each team in each round. As shown in Figure 1, a hula‐hoop displaying all the letters of the alphabet was used as a visual and functional aid. The contestant currently playing held the hula‐hoop to be distinguished from the other team members. Definitions were read aloud by an instructor in a clear and comprehensible manner, beginning with the letter A and proceeding sequentially through the alphabet. Contestants were required to identify the correct term corresponding to each definition. A correct answer allowed the team to continue; if a contestant passed or answered incorrectly, the timer was paused and control passed to the opposite team. When play resumed, the team continued from the letter immediately following the one on which they had previously passed or erred. Play continued until teams completed all 26 questions or exhausted their allocated time. If one team ran out of time, the other continued playing alone, with the option to pass, although the timer no longer stopped for passes. The winning team was determined by the highest number of correct answers. In the event of a tie, the team that completed their set of questions faster won. Figure 2 represents a schematic representation of the game‐based educational activity.

### 2.4 Data Collection and Analysis

Data were collected to assess both knowledge acquisition and student satisfaction resulting from the educational activity.

To assess knowledge retention, students completed a test at three time points: 2 weeks, 1 month and 1 year after the educational activity. The test consisted of a series of open‐ended, definition‐based questions, requiring students to write the exact term as listed in the glossary. The same test was administered to both first‐ and second‐year students. A trained evaluator, blinded to participant identity and group allocation, scored all written responses.

Additionally, to evaluate knowledge acquisition in relation to traditional educational methods, data were retrieved from the student cohort that preceded the implementation of the educational strategy. In this retrospective control group, students were provided with the *Glossary of Endodontic Terms* (AAE) and were assessed 1 month later using the same written test format described above.

To evaluate satisfaction, students were invited to complete an anonymous web‐based survey after the educational activity. The survey was designed using Google Forms and was adapted from a satisfaction survey used in a previous study (Arias et al. 2017). It included two sets of multiple‐choice items rated on a 5‐point Likert scale: one set focused on the impact of the content (five items) and the other on the instructional methods (five items). Additionally, the survey included four questions assessing overall satisfaction on a scale from 0 (the lowest) to10 (the highest level of satisfaction). Table 1 presents the specific items and questions included in these sections of the survey (as well as summaries of the participants' responses). It also contained four open‐ended questions inviting participants to provide qualitative feedback regarding the most and least favourable aspects of the activity, as well as suggestions for improving the learning of terminology and enhancing the overall experience. The survey link was distributed via email with a request to complete the questionnaire voluntarily.

**TABLE 1:** Satisfaction survey and responses from participants.

Set	ITEMS (Scale: 5‐point likert)	Cohort	% of responses				
			Strongly disagree	Disagree	Neither agree nor disagree	Agree	Strongly agree
Impact of the content	Content was adequate	First‐year	0	0	0	0	100
		Second‐year	0	0	16.7	16.7	66.6
	Content was relevant	First‐year	0	0	0	0	100
		Second‐year	0	0	0	16.7	83.3
	The concepts addressed were in line with the aim of the activity	First‐year	0	0	0	16.7	83.3
		Second‐year	0	0	0	50	50
	I have learned some terms that I did not know previously	First‐year	0	0	0	0	100
		Second‐year	0	0	0	16.7	83.3
	The content served to foster the use of endodontic terms	First‐year	0	0	0	16.7	83.3
		Second‐year	0	0	0	33.4	66.6
Instructional method	The method helped to keep my attention	First‐year	0	0	0	0	100
		Second‐year	0	0	0	0	100
	I learnt more with this method than with a more conventional format	First‐year	0	0	16.7	33.3	50
		Second‐year	0	0	33.3	16.7	50
	The method helped to increase my interest for the learning of endodontic terms	First‐year	0	0	16.7	16.7	66.6
		Second‐year	0	0	16.7	33.3	50
	The method helped to foster the use of endodontic terms	First‐year	0	0	0	33.4	66.6
		Second‐year	0	0	16.7	0	83.3
	The instructor did her best to make the activity interesting	First‐year	0	0	0	0	100
		Second‐year	0	0	0	0	100

Set	ITEMS (scale: numerical (0–10))	Mean (SD)	
		First‐year students	Second‐year students
Overall satisfaction	Overall satisfaction with the activity	10	9.67 (0.8)
	Overall quality of instruction	9.67 (0.8)	9.5 (0.8)
	Overall usefulness of the activity	9.33 (1.2)	9.83 (0.8)
	Overall organisation	9.5 (0.8)	9.83 (0.4)

*Note:* Summaries of results for overall satisfaction of students with the educational strategy [mean (SD)] and perceptions related to the impact of the content and the instructional method (percentage of responses per category—first‐year students/second‐year students).

Scores were recorded and analysed with IBM SPSS Statistics 28.0.1.1. Descriptive statistics were calculated for both test results and satisfaction scores. Due to violation of the assumption of normality, test scores and numerical satisfaction ratings (0–10 scale) were compared between cohorts using the non‐parametric Mann–Whitney *U* statistical test. Similarly, 1‐month test scores from the game‐based educational cohorts were compared to those of the retrospective control group using the non‐parametric Mann–Whitney *U* test. Comparison of test performance over time (2 weeks vs. 1 month/1 year) was analysed with the Wilcoxon‐signed rank paired test. Responses to the Likert scale items assessing the impact of content and instructional methods were compared between cohorts using the ordinal chi‐squared test.

## 3 Results

A total of 12 students participated in the study (16.7% males and 83.3% females).

### 3.1 Test Results

Mean test scores [standard deviation (SD)] for first‐year students were 9.17 (1.2), 9.17 (0.8) and 8.67 (1) at 2 weeks, 1 month and 1 year post‐intervention, respectively; and 9 (1), 9.33 (0.6) and 8 (0.7) for second‐year residents at the same intervals. No statistically significant differences were observed between the two cohorts at any time point. Likewise, within‐group comparisons showed no significant changes in scores over time, regardless of the interval between the educational activity and the assessment (*p* > 0.05). Students who participated in the game‐based educational intervention achieved significantly higher scores 1 month after the activity than those in the retrospective control group [mean (SD) = 7.25 (0.5)] who were assessed 1 month after receiving the glossary of endodontic terms (*p* < 0.01).

### 3.2 Satisfaction Survey

No significant differences were detected in the level of satisfaction between first‐ and second‐year students (*p* > 0.05). The 12 students completed the questionnaire demonstrating high satisfaction for each independent category and with the overall organisation, usefulness and quality of the educational strategy. Open‐ended questions provided interesting feedback. Table 1 shows separate summaries of results for first‐ and second‐year students for overall satisfaction of students with the educational strategy [mean (SD)] and perceptions related to the impact of the content and the instructional method (percentage of responses per category).

Open‐ended questions provided valuable feedback. Students particularly appreciated the engaging nature of the instructional method and emphasised how this approach is likely to enhance their retention of endodontic terminology. Some representative comments were ‘It was a fun and different way to study; I would love to have more sessions like this’; ‘It encouraged teamwork and improved communication among us’; ‘Thanks to the game I'll be able to remember endodontic terminology more easily in the future’.

## 4 Discussion

This study investigated the potential utility of applying constructivist principles to a postgraduate endodontic programme in relation to teaching endodontic terminology. Gamified classroom dynamics transforms passive lessons into interactive sessions with visual and audio stimuli. In these sessions, students become active participants, while the instructor takes on the role of a facilitator, motivating students' interaction (Wang and Tahir 2020). This plays into the constructivist theory by promoting learners' autonomy, active engagement and knowledge construction through personal experience, with the teacher serving as a facilitator in the students' journey toward deeper understanding (Rohman and Fauziati 2022). In fact, motivation is influenced by factors such as control, challenge, curiosity and self‐determination. The use of game‐based strategies that track personal scores and rank participants enhance intrinsic motivation by encouraging students to perform well in competitive environments (Castro et al. 2019).

Contemporary students are strongly influenced by digital technology and tend to prefer interactive and technology‐enhanced learning environments over traditional methods (Shorey et al. 2021; Siyami et al. 2023). The results of the present study showed that students were able to recall challenging terminology, even the terms that they had failed to identify during the educational session, even 1 year after the game‐based learning activity. Furthermore, these students demonstrated a significantly higher level of knowledge acquisition compared to a prior cohort used as retrospective controls. Therefore, this research suggests that game‐based learning can effectively support educational plans as it increases students' engagement and their interest. This can be attributed to the fact that gamification provides a fun and entertaining incentive, leading to high student satisfaction (Rodrigez‐Andres et al. 2017; Sipiyaruk et al. 2018). However, the results of the study should be interpreted with caution, since this educational strategy was piloted within a postgraduate programme; and hence, with a small sample size. At the same time, the limited number of enrolled students made it feasible to conduct an initial evaluation and collect preliminary data for an adequate power analysis before implementing the strategy with undergraduate students in a future study.

Some other limitations were identified during the design of the educational activity. First, by including two different cohorts of students (first‐ and second‐year), the instructor needed to classify endodontic terminology into two different levels of difficulty, which was a challenging task. Second, the set of questions was built randomly, and students might have preferred the other set of questions. However, as demonstrated during the assessment, students benefited equally from questions assigned to their own team, to opposing teams, and even to other cohorts. In fact, efforts were made to ensure an equal representation of questions from each set in the tests.

The feedback received from the satisfaction survey was also very insightful. All students completed the survey. Notably, 91.7% of students rated their overall satisfaction as 10, and 100% and 83.3% of first‐ and second‐year participants (respectively) strongly agreed that they learned terminology that they did not know previously. When inquired about their best perceptions of the activity, students shared highly positive comments. One student described the activity as both didactic and fun, noting that it helped reinforce concepts and allowed them to learn from their own mistakes. Another student expressed surprise at the large amount of terminology covered in a single session, while another highlighted the eagerness to learn with the method and the enthusiasm of both participants and instructors. The students also reported that this pilot educational game was a valuable experience, helping them recall and apply knowledge while promoting teamwork. Finally, one student characterised the game‐based activity as clear, concise, and consistent.

The meta‐analysis published by Baptista and Oliveira (2019) identified that perceived usefulness, enjoyment and attitude were key predictors of engagement in gamified learning activities. Similarly, Krath et al. (2021) systematically reviewed the theoretical foundations of gamification and concluded that its effectiveness is linked to mechanisms such as feedback, autonomy and adaptive complexity, all of which are aligned with self‐determination and constructivist learning principles. Thus, broader theoretical principles of effective instructional design might explain the success of the present intervention and the high levels of satisfaction observed in the participants. However, dental‐specific research remains comparatively underrepresented. Most publications are centered on medical and nursing educational environments, with only 3.77% of reviewed articles studying gamification in dental education (Lee et al. 2025). Moreover, there is a high heterogeneity between published articles, mainly regarding games design and outcomes (Lee et al. 2025). Therefore, further studies are needed to confirm that gamification has enough benefits over other methods since educators might need to be trained before game‐based learning is institutionalised in educational programmes (Godwin‐Jones 2014).

On the other hand, the negative feedback primarily concerned the unexpected nature of the activity. In order to prevent students from preparing in advance, they were not informed about the activity beforehand, and the evaluation dates were deliberately omitted from the informed consent. This approach was clearly described in the protocol submitted to the IRB, which did not raise any objections and granted an exemption. Students likely expected the test to occur at the end of the academic year and might have achieved better results had they been explicitly informed about the activity beforehand or the evaluation schedule. However, from the authors' perspective, it seemed interesting to isolate the potential effect of the educational strategy. Moreover, given that terminology is essential for everyday interactions with both peers and patients, evaluating casual retention was deemed important. In fact, the results of the tests demonstrated a positive impact of the game‐based strategy in short‐ and long‐term retention of endodontic terminology. The questions included in the tests were drawn from the pool of those used during the session, so good results shortly after the activity were expected. Students learned thorough simple competition with their peers. A positive finding emerged in the follow‐up test 1 year after the initial activity, students also demonstrated retention over a longer period, indicating the lasting impact of the game‐based learning strategy. However, while the human need to self‐assess and compare to others can have a motivational effect in the learning experience, it can also be a negative motivator and have detrimental effects depending on the context (Krath et al. 2021).

Moreover, it must be acknowledged that participants' broader clinical and didactic experiences over the programme period may also have contributed to their knowledge acquisition. This limitation is common in gamification research where long‐term, isolated outcome assessment is challenging (Lee et al. 2025) and prevents attributing all learning gains solely to the gamified intervention. Future studies trying to isolate the effect of the intervention with extended follow‐ups are needed.

Although incorporating games as a learning method has proven significant advantages, it is still not a tool commonly used. This may be due to a lack of knowledge about its benefits and the substantial effort required to organise an educational game, which demands significant involvement from both students and instructors. However, despite the more complicated preparation process, the non‐presential workload for students could be reduced, as they may need less time to achieve the same learning outcome (Scott 2003).

The present study supports findings from previous research. Aubeux et al. (2020) conducted an endodontic‐themed escape game activity with undergraduate students, resulting also in high scores and high levels of satisfaction. Incorporating game‐based learning could potentially be a successful learning experience. However, the incorporation of such activities may not work equally well for all instructors. No formal evaluation of the instructors' performance or attitude towards gamification was conducted; however, the instructors who designed the present study also conducted the activity and have strong commitment with active learning methodologies. Victoroff and Hogan (2006) demonstrated that the personal qualities of the instructor such as approachability, enthusiasm and willingness to provide guidance and feedback, contribute to effective learning experiences. Additionally, specific instructor skills like checking in with students and employing an interactive teaching style might be key factors in successful learning. Given the overall satisfaction reported by students in the present and previous studies when using game‐based educational strategies, future studies should explore their potential further. Universities should also consider providing additional training for instructors to foster active student engagement through these methods.

## 5 Conclusions

The high test scores and positive satisfaction ratings observed in both student cohorts suggest that game‐based educational strategies offer an innovative and practical complement to traditional methods for memorising endodontic terminology.

## Author Contributions

Conceptualization: I.F.‐G., A.A.; methodology: I.F.‐G., P.R., A.A.; formal analysis: A.A.; investigation: I.F.‐G., P.R., A.A.; data curation: I.F.‐G., A.A.; writing – original draft preparation: I.F.‐G., P.R., A.A.; writing – review and editing: A.A.; supervision: A.A. All authors have read and agreed to the published version of the manuscript. All authors contributed to the present manuscript. This study was selected for presentation in the ESE Education Prize category in 2023.

## Conflicts of Interest

The authors declare no conflicts of interest.

## Data Availability

The data that support the findings of this study are available from the corresponding author upon reasonable request.
